# Hypothyroidism has a protective causal association with hepatocellular carcinoma: A two-sample Mendelian randomization study

**DOI:** 10.3389/fendo.2022.987401

**Published:** 2022-09-30

**Authors:** Likui Lu, Bangbei Wan, Lingjun Li, Miao Sun

**Affiliations:** ^1^ Institute for Fetology, the First Affiliated Hospital of Soochow University, Suzhou, China; ^2^ Reproductive Medical Center, Hainan Women and Children’s Medical Centre, Haikou, China; ^3^ Department of Urology, Central South University Xiangya School of Medicine Affiliated Haikou Hospital, Haikou, China

**Keywords:** hepatocellular carcinoma, hypothyroidism, Mendelian randomization, genome-wide association study, single-nucleotide polymorphisms

## Abstract

**Objective:**

Observational studies suggest an association between hypothyroidism and the risk of hepatocellular carcinoma (HCC), but the causality and direction of these effects are still inconclusive. We aim to test whether hypothyroidism is causally associated with the risk of HCC by using Mendelian randomization (MR) analysis.

**Methods:**

Single-nucleotide polymorphisms (SNPs) associated with hypothyroidism were screened *via* a genome-wide association study (GWAS) on 337,159 individuals of European descent (16,376 cases and 320,783 controls). The SNPs associated with thyroid-stimulating hormone (TSH) and free thyroxine (FT4) were selected from a GWAS of 72,167 individuals of European descent. Summary-level data for HCC (168 cases and 372,016 controls) were extracted from UK Biobank. An inverse-variance-weighted (IVW) method was used as the primary MR analysis. Sensitivity analyses were examined *via* MR-Egger regression, heterogeneity test, pleiotropy test, and leave-one-out sensitivity test. The assumption that exposure causes outcome was verified using the MR Steiger test.

**Results:**

Two-Sample MR analysis showed inverse associations between genetically predicted hypothyroidism and HCC risk (OR = 0.997, 95% CI, 0.995-0.999; *P* = 0.016). There were no statistical indications of heterogeneity among instruments (*P*-het = 0.667). Across five MR methods, genetically predicted hypothyroidism shows a consistent correlation with HCC. The leave-one-out analysis indicated that no single SNP changed the overall estimate (*P* = 0.016). In addition, the MR Steiger test revealed that hypothyroidism was causal for HCC and not the opposite (*P* = 0.000). Finally, there was no evidence for a direct causal effect of TSH level and FT4 level on HCC risk.

**Conclusion:**

Our results provide some that genetically determined hypothyroidism decreases the risk of HCC, although the size of the causal estimate is small. Further research is required to comprehend the mechanisms underlying this putative causative association, and follow-up clinical trials need to be conducted to establish whether inducing hypothyroidism could be beneficial for patients who are suffering from HCC. During future treatment of hypothyroidism, close attention to liver function may also be required to prevent a possible increased risk of HCC.

## Introduction

Hypothyroidism is a prevalent condition that affects 0.6% to 12% of women and 1.3% to 4% of males, with the highest prevalence among the elderly (1–4). Hypothyroidism can manifest as increased thyroid-stimulating hormone (TSH) serum levels, resulting in decreased thyroid hormone production [triiodothyronine (T3) and/or thyroxin (T4)]. In iodine-rich environments, the most prevalent causes of hypothyroidism are autoimmune disorders, such as Hashimoto’s thyroiditis.

Hypothyroidism may contribute to the development of cancer. Thyroid hormones and TSH can directly stimulate tumor formation *via* cell surface receptors, estrogen pathways, increased angiogenesis, and gene expression modification (5, 6). In addition, hypothyroidism is related to diabetes mellitus and cardiovascular disorders (7), both of which have been associated with elevated cancer risk. However, the epidemiological studies of the link between hypothyroidism and cancer risk are equivocal. Several studies have reported that increased risks of thyroid cancer (8) and decreased risks of breast cancer (8) were associated with hypothyroidism. However, in some other large cohorts, there was no association between hypothyroidism and cancer risk (9).

Nonetheless, interpretation of these findings is hindered by various issues, including a paucity of longitudinal research, variances in target populations, a lack of data on thyroid dysfunction treatments, the inclusion of prevalent cancer cases, and the possibility of reverse causality. Boursi et al. found that untreated hypothyroidism is related to an increased risk of colorectal cancer (CRC), but long-term thyroid hormone replacement is associated with a lower risk of CRC, and this protective association increases with a cumulative effect of medication (10). However, Wang et al. reported no significant correlation between thyroid hormone replacement therapy and the risk of breast cancer, and the correlation between hypothyroidism and the risk of breast cancer is related to the observed population (9).

Recent investigations have found correlations between hypothyroidism and additional cancer types, including liver cancer. Observational study results indicate hypothyroidism may be a risk factor for HCC (11–14). Even though they are helpful, these observational studies are susceptible to confounding factors, leading to inaccurate causal inferences (15). Therefore, randomized controlled trials are necessary to test whether the links found in observational studies can produce the expected health benefits.

Randomized studies on thyroid illness are challenging because large sample sizes and prolonged follow-up are required. Therefore, drawing the causal connection between thyroid disease and adverse outcomes becomes challenging. An efficient approach must be identified to determine whether or not the reported correlation between thyroid malfunction and adverse outcomes is a causal relationship. Mendelian randomization (MR) method has the potential to address this question in particular (16–19). Since genetic mutations are innate and are not affected by environmental factors, the MR research method using single-nucleotide polymorphisms (SNP) as an instrumental variable can well control the interference of confounding factors, similar to a randomized trial. Furthermore, genetic variation can affect outcomes, but outcomes cannot affect genes, so no inference of reverse causality can be drawn. MR is based on the following assumptions: the genetic instrument should be strongly associated with the exposure but not associated with confounders, and the genetic variant affects the outcome only through the risk factor (16). Causality can be established through a genetic instrument that fits all MR assumptions. If MR identifies a causal link with a risk factor, further clinical studies are necessary to be conducted to establish the efficacy of a treatment that targets the same risk factor. It is worth noting that in order to be conducted, clinical trials should meet certain requirements. First, the exposure factors found must be modifiable (such as some metabolites, etc.); Secondly, the intervention should not have serious adverse effects on the human body (requires a very careful assessment); finally, it must be ethical.

MR studies require tens of thousands of individuals because the effects of the investigated genetic instruments on risk factors are often modest. This strategy is viable for most thyroid-related outcomes due to the availability of results from large-scale genome-wide association studies (GWASs) (20) for numerous traits, which paved the way for adequately powered MR investigations. Based on MR method, Yuan et al. successfully predicted the negative causal effect of hypothyroidism on breast and thyroid cancer (21). Meanwhile, Yuan et al. found that there was no causal association between hypothyroidism and HCC (21). However, the hypothyroidism dataset used in Yuan’s study had a small sample size, which is also an important factor affecting the results of the MR analysis (22).

However, as larger the GWAS datasets become available, it is necessary to re-study whether it will lead to different results. To that end, we performed a two-sample MR to investigate the causal relationship between hypothyroidism and HCC. In addition, since hypothyroidism is closely associated with circulating TSH and free T4 (FT4) level, we also included TSH and FT4 in the MR analysis.

## Methods

### Study design

The summary-level data used to conduct the two-sample MR study was taken from the IEU Open GWAS database (https://gwas.mrcieu.ac.uk/), namely hypothyroidism (GWAS ID: ukb-a-77) and HCC (GWAS ID: ieu-b-4953). In addition, the summary-level data including TSH and FT4 was extracted from the Thyroid Omics Consortium (20). The relevant ethics committee authorized initial GWAS, and all subjects supplied informed permission. We received a waiver from the Ethics Committee of the First Affiliated Hospital of Soochow University, as all datasets used for this research were obtained from the public domain.

### Assumptions of Mendelian randomization study

**Figure 1:**
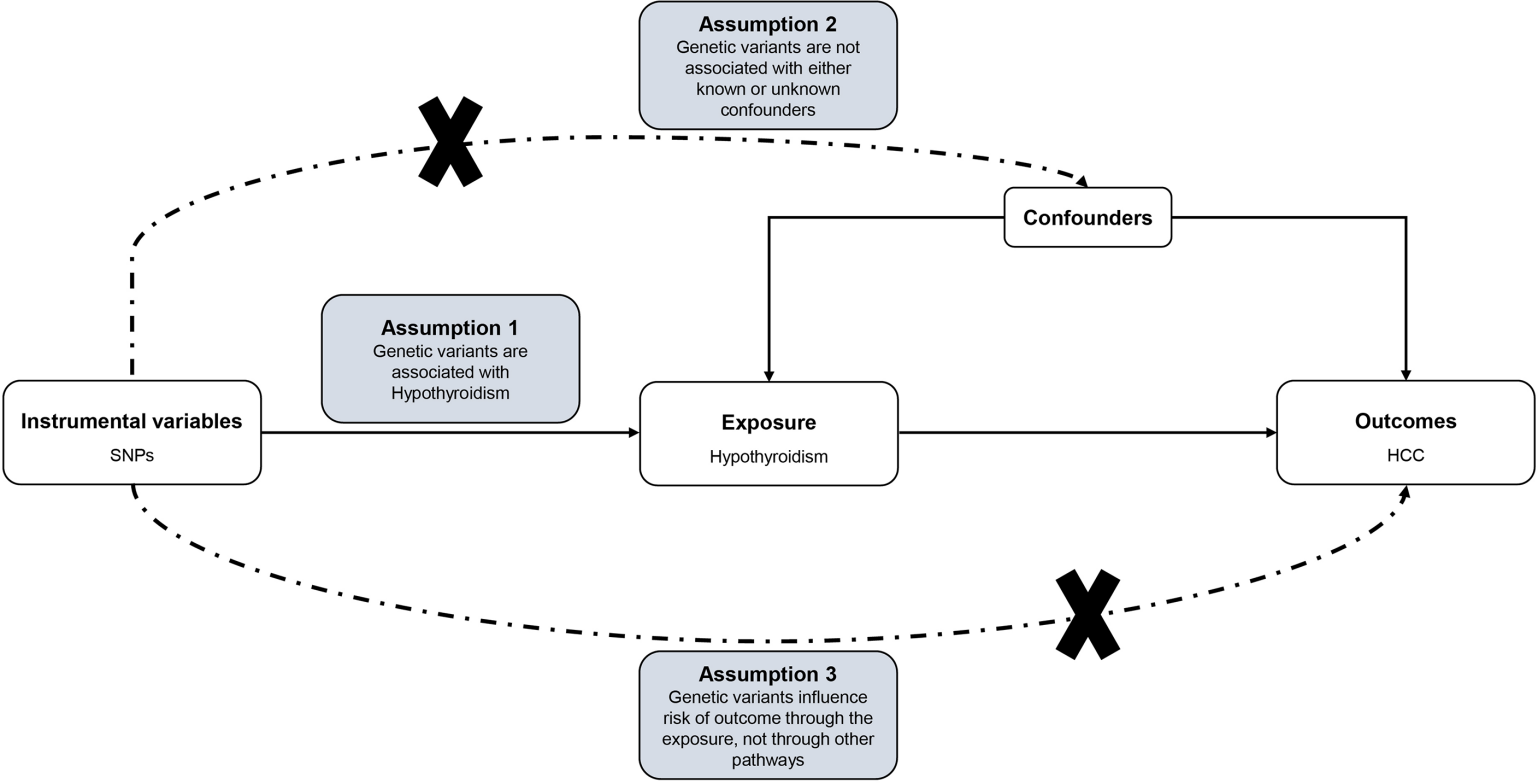
Directed acyclic graph of the MR framework investigating the causal relationship between Hypothyroidism and HCC. Instrumental variable assumptions: (1) the genetic instrument variables (GIVs) must be strongly associated with hypothyroidism; (2) the GIVs must not be associated with any potential confounder of hypothyroidism vs HCC relationship; (3) the GIVs should only affect the risk of HCC through hypothyroidism. SNPs, single-nucleotide polymorphisms; HCC, Hepatocellular carcinoma; MR, Mendelian randomization.

For causal estimates from MR studies to be valid, three main assumptions must be met: (1) the genetic instrument variables (GIVs) must be strongly associated with hypothyroidism; (2) the GIVs must not be associated with any potential confounder of hypothyroidism vs HCC relationship; (3) the GIVs should only affect the risk of HCC through hypothyroidism (ie, horizontal pleiotropy should not be present) (23, 24). The assumptions and design of the MR study are shown in **Figure 1**.

### Data on exposures and outcome

Based on the GWAS of European ancestry, we identified independent SNPs associated with hypothyroidism, TSH and FT4 at a genome-wide significant level (*P* < 5×10^-8^). Independence of SNPs was assessed using stringent criteria (*r^2^* ≤ 0.001; clumping window, 10 000 kb). All included SNPs and their details were shown in **Tables S1–S3**. In addition, the GWAS summary statistics data of HCC of European ancestry (372, 184 individuals) were downloaded *via* the IEU Open GWAS database. Participants in the hypothyroidism research program were not screened for HCC. Instrument strength for each SNP in MR was estimated with an approximated F statistic using the following formula: F = *R ^2^* (N-2)/(1-*R ^2^*), where *R^2^* is the proportion of the variance of the hypothyroidism explained by each genetic variant, and N is the sample size of the GWAS for the hypothyroidism. *R^2^* for the instrument variants was estimated using the following formula: 2^∗^EAF^∗^(1 −EAF)^∗^ *β* (25), where EAF is the effect allele frequency, and *β* is the estimated genetic effect on hypothyroidism (26). As *β* coefficients and standard errors were not available in the summary-level data, we used methods described by Burgess and Davey Smith to calculate them (27).

### Two-sample MR

Two-sample MR was performed using the genetic data extracted from the GWAS summary statistics. We employed the TwoSampleMR package (version 0.5.6) in R (version 4.1.2) (28) to integrate and analyse data. In this study, the inverse-variance-weighted (IVW) (29) method was used as the primary causal effect estimation method to calculate the combined effect of all SNPs. At the same time, MR-Egger (30), weighted median (31), simple mode (32), and weighted mode (32) methods were adopted to test the reliability and stability of the results. Two-sample MR analysis may have heterogeneity due to differences in analysis platforms, experimental conditions, inclusion populations, and SNPs, thereby biasing estimates of causal effects. Therefore, the main IVW and MR-Egger methods were tested for heterogeneity in this study. If the *P*-value is greater than 0.05, it is considered that there is no heterogeneity in the included instrumental variables, and the influence of heterogeneity on the estimation of causal effects can be ignored. One of the assumptions of MR analysis is that instrumental variables can only affect outcomes through exposure. If a GIV directly affects outcomes without affecting exposure, it violates the idea of MR. Therefore, it is necessary to test whether there is pleiotropy in the causal inference between exposure and outcome. The intercept of the Egger model can be used for the statistical test of pleiotropy, where a deviation from 0 denotes the presence of directional pleiotropy (33). In this study, the *P*-value of the pleiotropy test is used to measure whether there is pleiotropy in the analysis. If *P* > 0.05, the possibility of pleiotropy in the causal analysis is considered weak, and its impact can be ignored. The presence of pleiotropy in the analysis was also determined in this study using the MR-pleiotropy residual sum outlier (MR-PRESSO) (34). This study also used the leave-one-out method and heterogeneity test for sensitivity analysis. The directionality that exposure causes outcome was verified using the MR Steiger test, *P* < 0.05 was regarded as statistically significant. The *a priori* statistical power was calculated according to Brion et al. (35).

## Results

All 71 independent genetic variants associated with hypothyroidism were available in the summary statistics for HCC. The F statistic of these SNPs was greater than 10 (range, 38-175; mean, 57) in hypothyroidism, indicating a low risk of weak-instrument bias (**Tables S1**, **Figure S1**). In addition, all 35 and 12 independent genetic variants associated with TSH and FT4 were available in the summary statistic for HCC, respectively. Detailed information on these SNPs can be found in **Tables S2** and**S3**.

**Figure 2:**
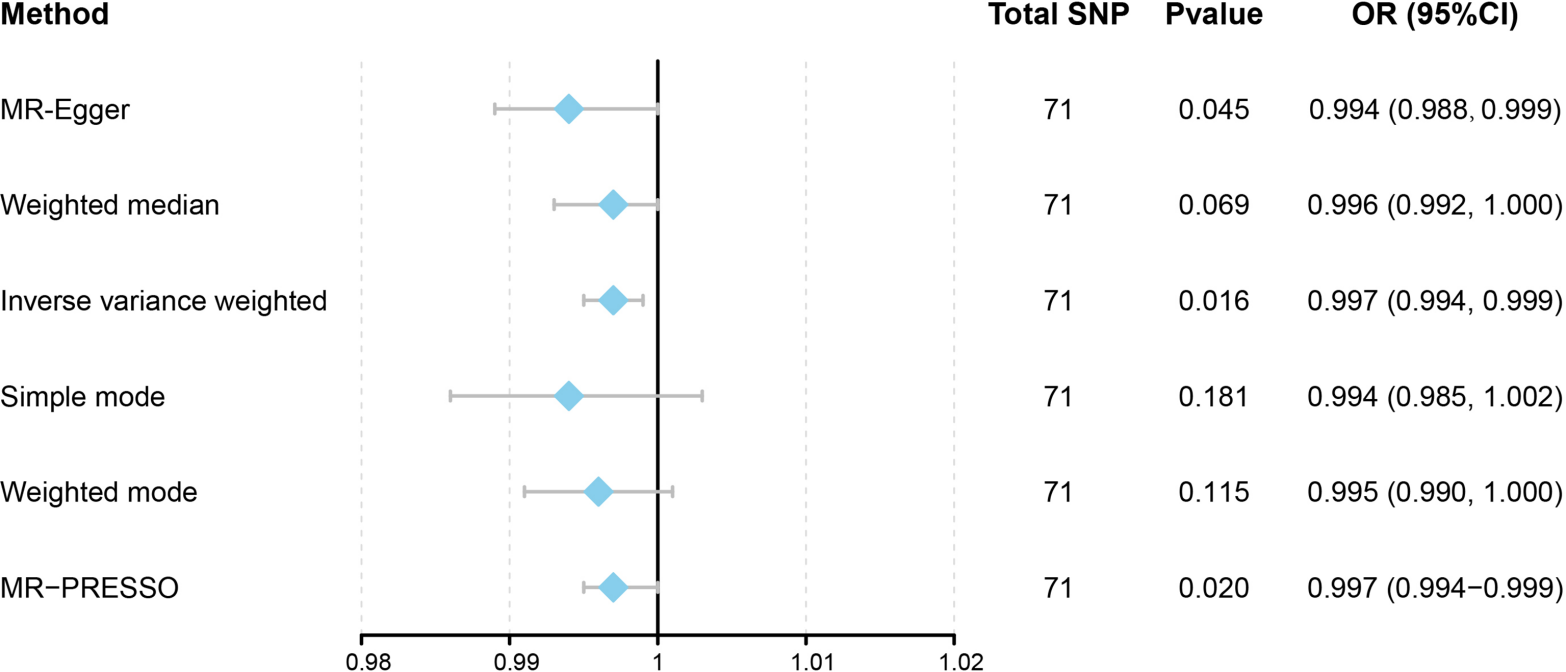
Forest plot to visualize causal effects of variation in Hypothyroidism on HCC. Presented odds ratios (OR) and confidence intervals (CI) correspond to the effects of Hypothyroidism on HCC. The results of Mendelian Randomization (MR) analyses using various analysis methods (MR-Egger, Weighted median, Inverse variance weighted, Simple mode, and Weighted mode) are presented for comparison. Total single-nucleotide polymorphism (SNP) indicates the number of genetic variants used as instruments for MR analysis.

**Figure 3:**
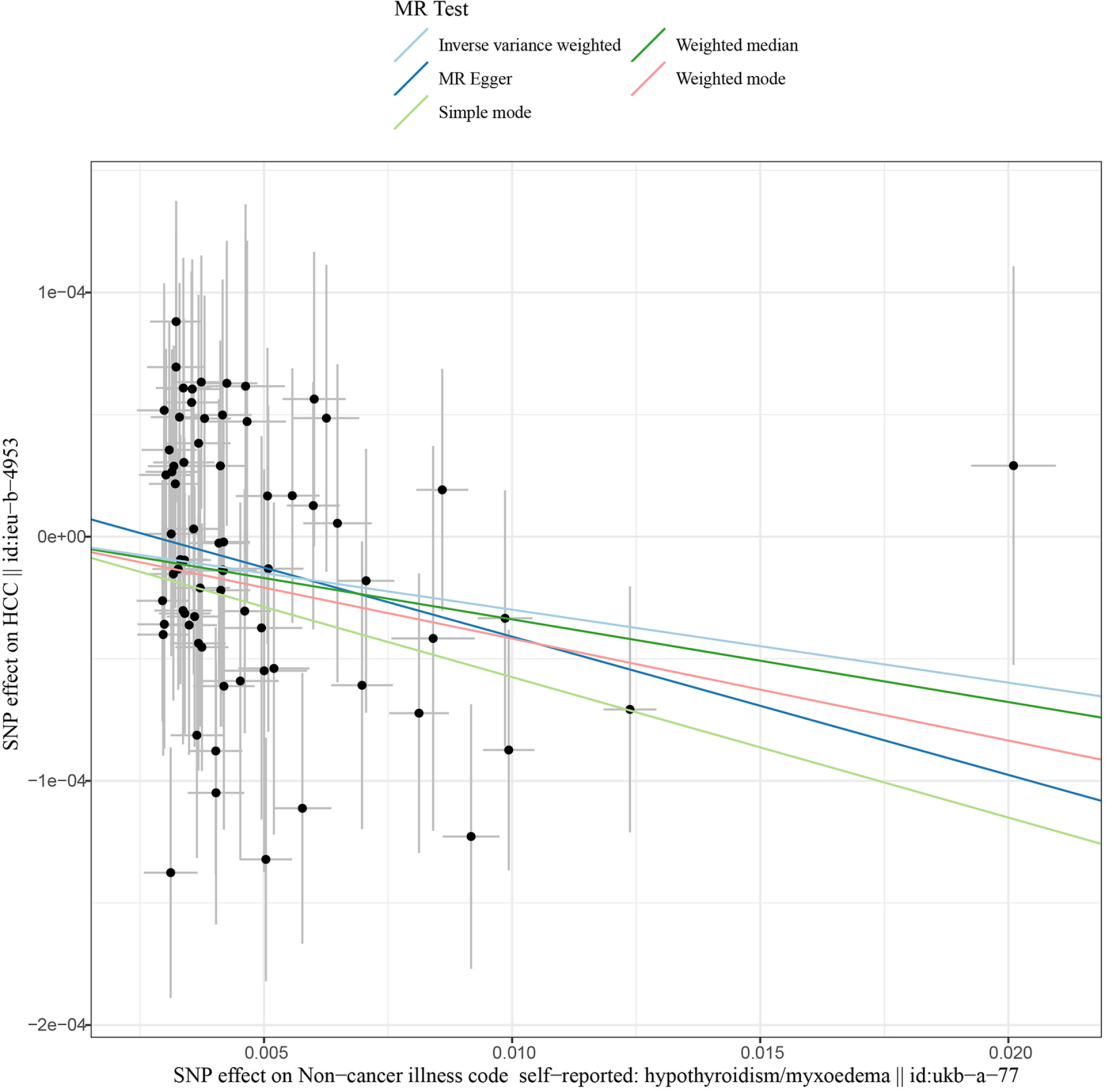
Scatter plots of Hypothyroidism with the risk of HCC. Scatter plot demonstrating the effect of each hypothyroidism-associated SNP on HCC on the log-odds scale. The slopes of each line represent the causal association for each method. MR, Mendelian randomization; SNP, single-nucleotide polymorphism; HCC, Hepatocellular carcinoma.

**Figure 4:**
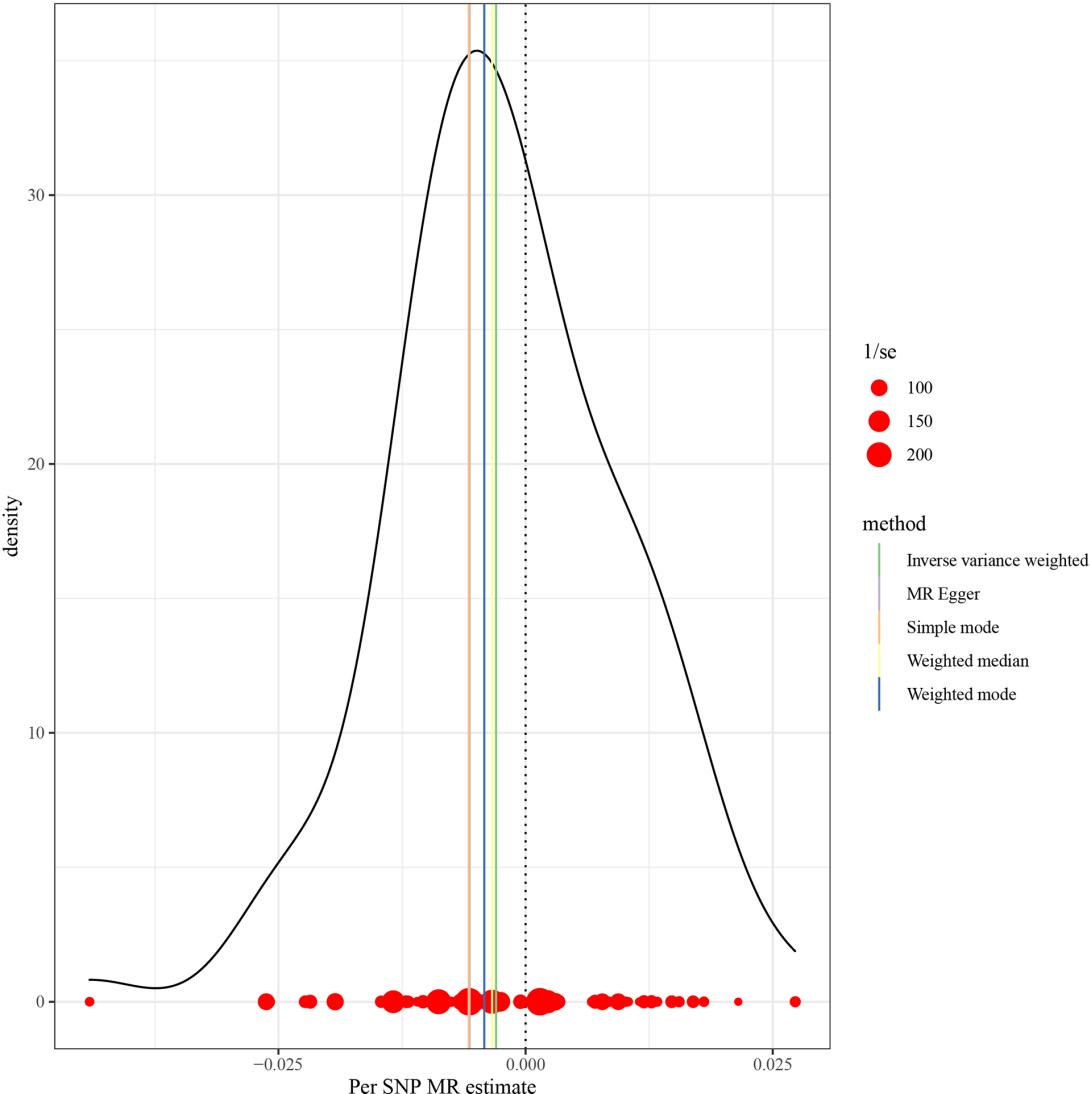
MR density plots to visualize the overall heterogeneity of MR estimates for the effect of Hypothyroidism on HCC. MR, Mendelian randomization; SNP, single-nucleotide polymorphism; HCC, Hepatocellular carcinoma.

**Figure 5:**
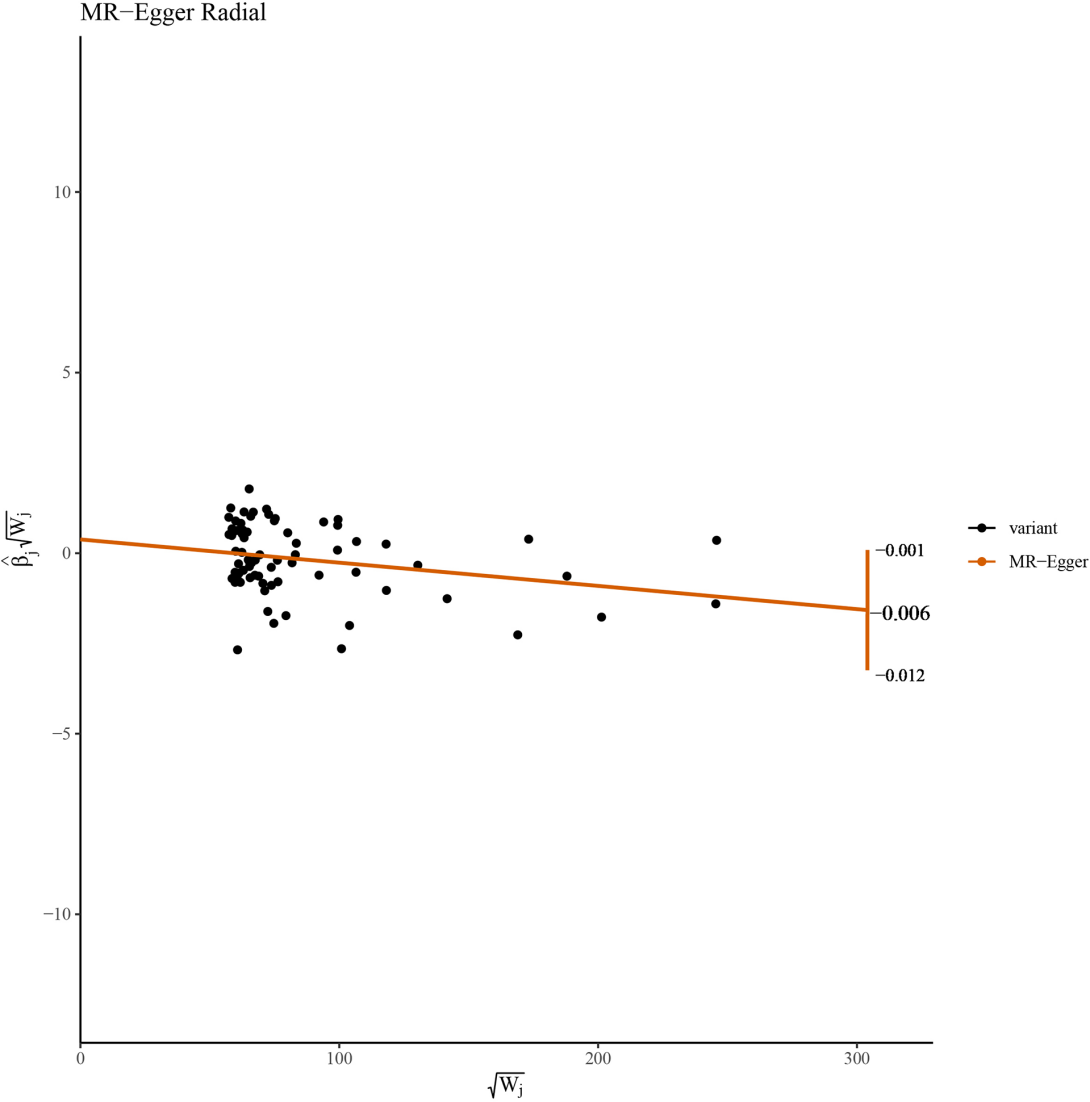
Radial plots of the MR−Egger test analyzed the outlier SNPs.

**Figure 6:**
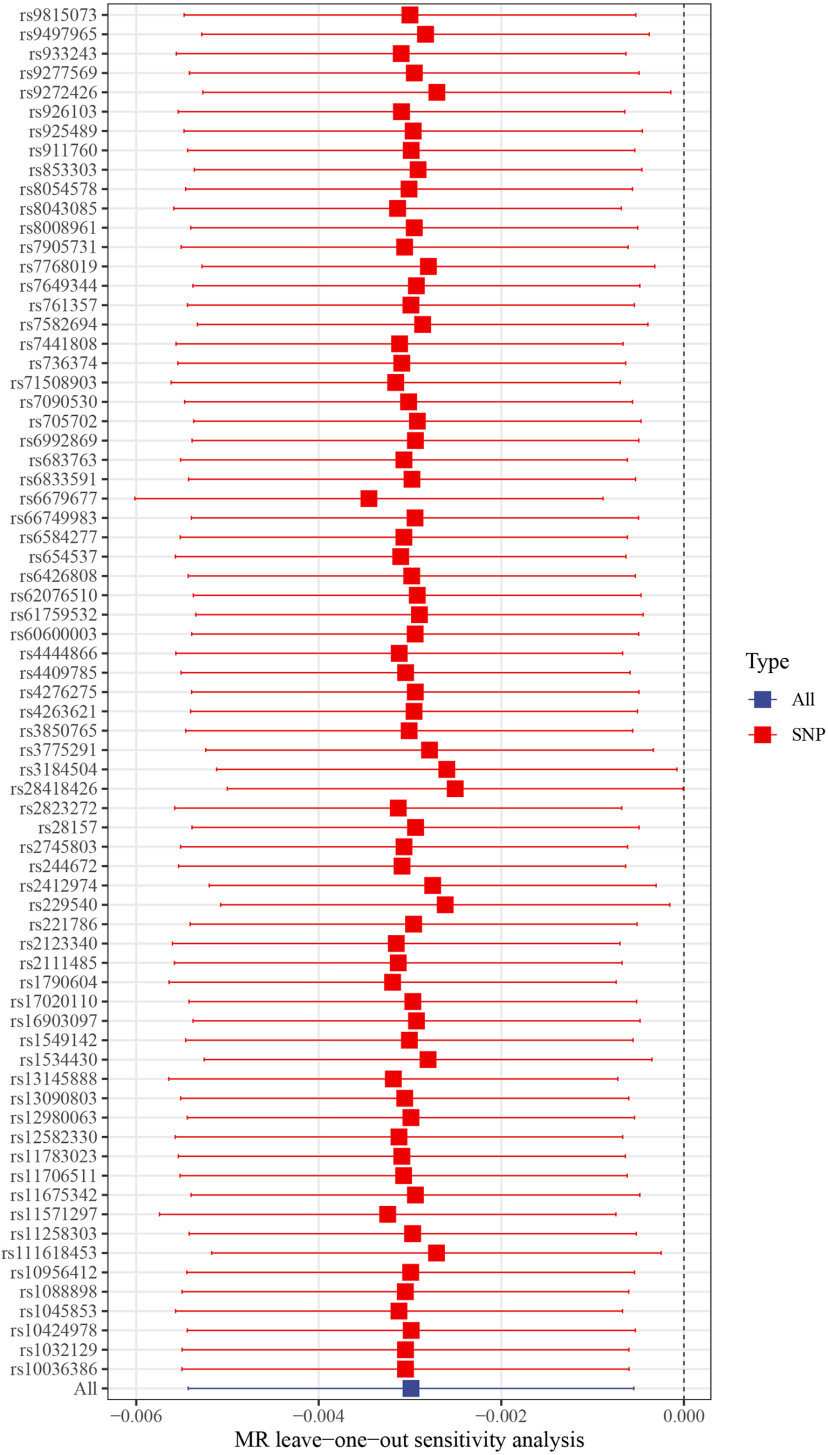
Leave-one-out plots of hypothyroidism with the risk of HCC. Leave-one-out analysis for IVW MR of hypothyroidism on HCC in summary-level analyses. SNP, single-nucleotide polymorphism; HCC, Hepatocellular carcinoma; MR, Mendelian randomization.

This work showed that genetically predicted hypothyroidism was inversely associated with HCC; the odds ratios (OR) was 0.997 (95% CI, 0.994 - 0.999; p = 0.016) in the IVW analysis (**Table 1** and **Figure 2**). No statistical evidence of heterogeneity was found across instruments (*P*-het = 0.666). Genetically predicted hypothyroidism showed a broadly consistent association with HCC across the different MR methods (**Table 1s**), although the results of weighted median and mode-based methods did not reach statistical significance. The Scatter plots also showed that the slopes of the results between hypothyroidism and HCC analyzed by different methods are all negative, and the consistent correlation trend indicates that our analysis results are very reliable (**Figure 3**). In addition, density plots showed that the estimated effect values of most SNPs are distributed in a certain area, suggesting that there was no significant heterogeneity in our study (**Figure 4**). The intercept term estimated from MR-Egger was centred at the origin (*P*-intercept = 0.2365), suggesting that directional pleiotropy did not influence the results. No outlier SNP was identified that led to increased pleiotropy in the overall MR estimate by MR-PRESSO analysis and MR-Egger test (**Figure 5**). In addition, no single SNP affected the overall estimate as demonstrated by the leave-one-out analysis (*P* = 0.016) (**Figure 6**). Moreover, the SNPs explained 1.75% of the variance of hypothyroidism.

**Table 1:** MR Results of Hypothyroidism on Risk of HCC.

Exposure	Method	No. of SNPs	OR (95% CI)	P	P-het	P-intercept	P-global
	MR-Egger	71	0.994 (0.988, 0.999)	0.045	0.673	0.283	
	Weighted median	71	0.996 (0.992, 1.000)	0.069			
Hypothyroidism	IVW	71	0.997 (0.994, 0.999)	0.016	0.666		
	Simple mode	71	0.994 (0.985, 1.002)	0.181			
	Weighted mode	71	0.995 (0.990, 1.000)	0.115			
	MR-PRESSO (raw)	71	0.997 (0.994-0.999)	0.020			0.669

HCC, Hepatocellular carcinoma; IVW, inverse variance weighted; MR, Mendelian randomization; OR, odds ratio; P-het, P value for heterogeneity using Cochran Q test; P-intercept, P value for MR-Egger intercept; SNP, single-nucleotide polymorphism.

The causal assumption of hypothyroidism and HCC was verified *via* the MR Steiger test, and the result showed hypothyroidism’s influence on HCC was the correct causal direction (*P* = 0.000). In addition, given a type 1 error of 5%, and OR for HCC was 0.997 in genetically instrumented hypothyroidism use, there was 5% power to detect the causal association between hypothyroidism and HCC.

In addition, in the MR analysis using TSH and FT4 as exposures, there was no evidence for a direct causal effect of TSH level (OR = 1.000, 95% CI = 0.999-1.000, *P* = 0.566) and FT4 level (OR = 1.000, 95% CI = 0.999-1.000, *P* = 0.433) on HCC risk (**Figures S2**, **S3**). The SNPs explained 6.35% and 2.86% of the variance of TSH and FT4, respectively. Moreover, power analysis showed that there was 5% power to detect the causal association between TSH, FT4 and HCC.

## Discussion

In the present study, we used the MR approach to estimate the causal effect of hypothyroidism on risk for HCC. We observed that genetically predicted hypothyroidism was inversely associated with HCC.

Hypothyroidism is a common thyroid hormone shortage that’s easily recognized and controlled, but can be fatal if left untreated. The concept of hypothyroidism is based on biochemical reference ranges and is debated. Hypothyroidism symptoms range from life-threatening to none. Most observational studies have found that hypothyroidism was related to a low risk of breast cancer (36, 37). Furthermore, the inverse association between hypothyroidism and breast cancer has been verified by MR analysis (21). Although the researchers also analyzed the causal relationship between hypothyroidism and many other cancers simultaneously, they found no solid evidence. However, as the number of cases included changes, it is crucial to reevaluate if this will result in different findings.

Since thyroid hormones have a role in the cell metabolism of the whole body, it is not surprising that the liver may also be affected by hypothyroidism. Despite this, the link between the liver and the thyroid gland is frequently disregarded, and thyroid function is rarely evaluated in patients with liver disorders and vice versa. Multiple studies have found that thyroid hormone may have a significant role in the etiology of a number of liver illnesses, including nonalcoholic fatty liver disease (NAFLD) (38) and fibrosis (39), which may evolve into cirrhosis and HCC. According to Bruck et al., pharmacologically induced hypothyroidism hastens the resolution of liver fibrosis in rats (40). Similarly, Oren et al. discovered that induced hypothyroidism could delay the development of cirrhosis in a rat model, whereas hyperthyroidism can exacerbate it (41). In addition, Gionfra et al. demonstrated that elevated rT3 might contribute to tumor cell growth (42). rT3 is a thyroid hormone frequently neglected in the past, but its function has received increasing attention in recent years. These findings imply that aberrant thyroid hormone levels are strongly associated with the development of liver illness, which may ultimately lead to the development of HCC, and that hypothyroidism may play a protective role against these conditions.

HCC is the most prevalent primary liver cancer and the ninth greatest cause of cancer-related mortality in the United States (43). Many risk factors are closely related to the occurrence and development of HCC. Cirrhosis is an essential risk factor for developing HCC, regardless of its cause. Moreover, observational studies have revealed that either overt or subclinical hypothyroidism is associated with a poor prognosis for advanced HCC (11). Long-term hypothyroidism (more than three years duration) was related to a significantly elevated risk (from 2.1 to 2.9-fold) of HCC, especially in women, independent of other well-known risk factors, such as HCV infection and diabetes (13). These findings indicated that hypothyroidism might be an extra risk factor for HCC. Although the mechanisms by which hypothyroidism can promote the development of HCC are unknown, susceptibility to HCC may be increased by Nonalcoholic Steatohepatitis (NASH), which is in turn facilitated by hyperlipidemia, decreased fatty acid oxidation, insulin resistance, and lipid peroxidation, all of which are frequently observed in hypothyroidism (13). Patients with HCC of unclear cause appear to have a significantly higher likelihood of hypothyroidism than patients with HCV-related HCC and controls (12).

Accordingly, we speculate that there may be a positive causal relationship between hypothyroidism and HCC. However, to our surprise, we found an inverse association between genetically predicted hypothyroidism and HCC using the two-sample MR approach. MR-Egger regression analysis showed no directional pleiotropy influenced the results. Furthermore, MR-PRESSO analysis, MR-Egger test, and leave-one-out analysis indicated no single SNP affected the overall MR estimate. Given the data above, the dependability of this negatively linked causal relationship is pretty strong. Although Yuan et al. found no causal association between hypothyroidism and HCC risk, this may be related to the fewer thyroid dysfunction cases (72,167 European-descent individuals) included in SNP screening (21); however, SNPs associated with hypothyroidism were selected from a GWAS analysis with up to 337,159 European-descent individuals in our study. Notably, after expanding the sample size, the results of present study showed that there was a negative causal relationship between hypothyroidism and HCC (OR = 0.997, 95% CI, 0.995-0.999) and there was a statistical difference (*P* = 0.016), which was different from the results of Yuan et al. (OR = 0.82, 95% CI, 0.55-1.23; *P* = 0.336) (21). Moreover, due to the fact that definition of hypothyroidism varies (based on more or less stringent TSH cut-offs and subjective symptoms). An MR study using continuous exposures (TSH and FT4) is deemed necessary in this context. Data showed that TSH level (OR = 1.000, 95% CI = 0.999-1.000, *P* = 0.566) and FT4 level (OR = 1.000, 95% CI = 0.999-1.000, *P* = 0.433) have no significant causal relationship with HCC risk. One possible reason for the lack of statistical significance in the results of TSH and FT4 is the problem of sample size. The sample size of TSH and FT4 exposure is 72,167, and the sample size needs to be further expanded in the future. In addition, the results of TSH and FT4 analysis may not have negated our results because the possible protective effect of hypothyroidism on HCC may not be achieved through thyroid function-related hormones (including TSH and FT4), and the specific mechanism remains to be further studied. Finally, it is also very important that the TSH and FT4 datasets may be derived from normal populations, so the measured TSH and T4 may be in the normal range, while the TSH or FT4 levels in hypothyroidism populations are likely not in this normal range, and the biological effects of abnormal TSH or FT4 are not well understood.

A major strength of this study is that it employs stringent quality control conditions and analysis methodologies, evaluates causal effects using a range of models, and produces dependable and steady research results. In addition, a greater sample size was added for MR analysis. In addition, we validated the relationship between hypothyroidism and the risk of developing HCC, thereby verifying the genetic instruments used to diagnose hypothyroidism.

Because our research was only conducted on people of European descent, there is a possibility that the results cannot be generalized to people of other ancestries, which means that the implications of our findings should be interpreted with caution. Another potential restriction is the absence of sex-stratified summary information on instrument exposure for hypothyroidism, which is more frequent in women than in males. In addition, the statistical power of this study is not very satisfactory. Given a type 1 error of 5%, and odds ratio (OR) for HCC was 0.997 in genetically instrumented hypothyroidism use, there was 5% power to detect the causal association between hypothyroidism and HCC. This may indicate a weak correlation between the hypothyroidism and HCC. The main reason for this result may be that there are too few HCC cases in the outcome dataset, and sample size needs to be expanded to further confirm the exact causal relationship between hypothyroidism and HCC. However, the power of a a MR study depends on the sample size and the strength of the association between GIVs and risk factors (22). The calculated F-statistics of all included SNPs are greater than 10, indicating that our causal inferences using these instrumental variables still have certain credibility and will not be disturbed by weak instrumental variables (22). Moreover, since there are no datasets on different types of hypothyroidism, such as overt and subclinical hypothyroidism, their effects on HCC may be different (because of different thyroid hormone levels). Therefore, when this study explores the relationship between hypothyroidism and HCC, the population diagnosed with hypothyroidism may have both overt hypothyroidism and subclinical hypothyroidism, which ultimately affects the inference of the directionality of the causal relationship between hypothyroidism and HCC. Finally, due to the fact that definition of hypothyroidism varies. An MR study using continuous exposures (TSH, FT4) is deemed necessary. However, due to the limited public summary statistic data supporting these exposures, it is difficult to interpret the effects of these factors.

In conclusion, using summary data from the UK Biobank, our results provide some evidence that hypothyroidism might be causally associated with HCC, using two-sample MR to support stronger causal inference, although the size of the association is small. However, due to the low statistical power of the current study, to avoid the possible adverse effects of causal inference errors, the sample size needs to be expanded to further verify the causal relationship between hypothyroidism and HCC. Furthermore, more research is needed to determine how this possible cause-and-effect link works, and follow-up clinical trials are required to see if causing hypothyroidism could be helpful for HCC patients. During future treatment for hypothyroidism, keeping a close eye on liver function may also be necessary to prevent an increased risk of HCC.

## Data availability statement

The datasets presented in this study can be found in online repositories. The names of the repository/repositories and accession number(s) can be found in the article/**Supplementary Material**.

## Ethics statement

The studies involving human participants were reviewed and approved by Ethics Committee of the First Affiliated Hospital of Soochow University. The patients/participants provided their written informed consent to participate in this study.

## Author contributions

Study conception and design: LLu, BW; Analyses: BW; Draft: LLu; Supervision: MS and LLi; Interpretation of results, critical editing, and manuscript approval: all authors.

## Funding

This work was supported by grants from the National Key R&D Program of China (2019YFA0802600), the National Natural Science Foundation of China (81974244), the Hainan Province Clinical Medical Center (QWYH202175), the Research and Cultivation Fund of Hainan Medical University (HYPY2020015), and the Postgraduate Research & Practice Innovation Program of Jiangsu Province (5832013521).

## Conflict of interest

The authors declare that the research was conducted in the absence of any commercial or financial relationships that could be construed as a potential conflict of interest.

## Publisher’s note

All claims expressed in this article are solely those of the authors and do not necessarily represent those of their affiliated organizations, or those of the publisher, the editors and the reviewers. Any product that may be evaluated in this article, or claim that may be made by its manufacturer, is not guaranteed or endorsed by the publisher.

## Supplementary material

Supplementary Figure 1 F statistic of included SNPs associated with hypothyroidism in MR analysis. The F statistic of those SNPs was greater than 10 (range, 38-175; mean, 57) in hypothyroidism.

Supplementary Figure 2 Forest plot to visualize causal effects of variation in TSH on HCC. Presented odds ratios (OR) and confidence intervals (CI) correspond to the effects of TSH on HCC. The results of Mendelian Randomization (MR) analyses using various analysis methods (MR-Egger, Weighted median, Inverse variance weighted, Simple mode, and Weighted mode) are presented for comparison.

Supplementary Figure 3 Forest plot to visualize causal effects of variation in FT4 on HCC. Presented odds ratios (OR) and confidence intervals (CI) correspond to the effects of FT4 on HCC. The results of Mendelian Randomization (MR) analyses using various analysis methods (MR-Egger, Weighted median, Inverse variance weighted, Simple mode, and Weighted mode) are presented for comparison.

## Abbreviations

HCC, hepatocellular carcinoma; MR, mendelian randomization; SNPs, single-nucleotide polymorphisms; GWAS, genome-wide association study; IVW, inverse-variance-weighted; T3, triiodothyronine; T4, thyroxin; CRC, colorectal cancer; GIVs, genetic instrument variables; NAFLD, nonalcoholic fatty liver disease.
