# Supplementary figures and images for: Hypothyroidism has a protective causal association with hepatocellular carcinoma: A two-sample Mendelian randomization study
Source: Front Endocrinol (Lausanne). 2022 Sep 30;13:987401. doi: 10.3389/fendo.2022.987401 (PMC9562779; doi:10.3389/fendo.2022.987401)

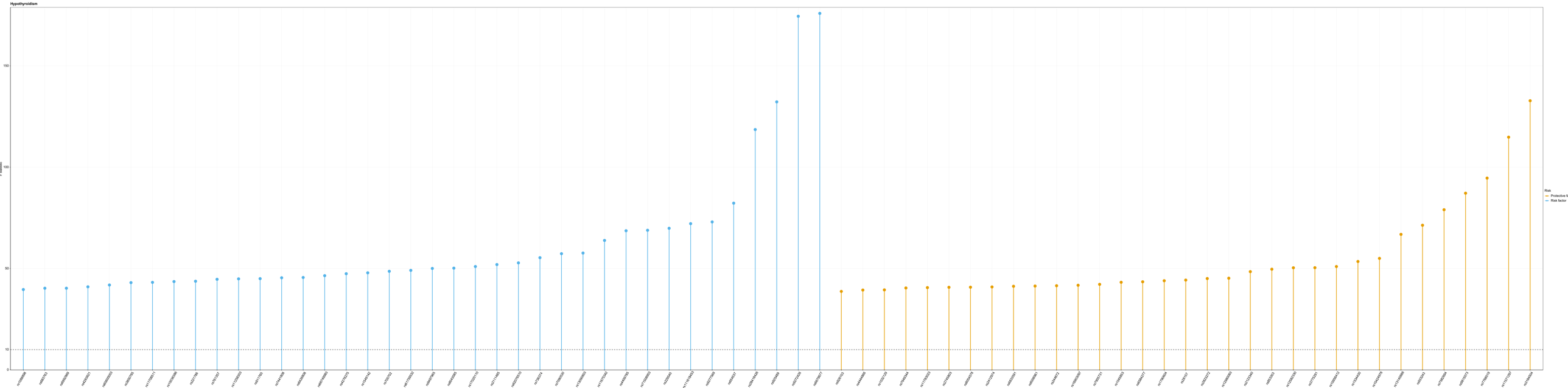

Supplement: Supplementary Figure 1 — F statistic of included SNPs associated with hypothyroidism in MR analysis. The F statistic of those SNPs was greater than 10 (range, 38-175; mean, 57) in hypothyroidism. [file DataSheet_1.pdf]

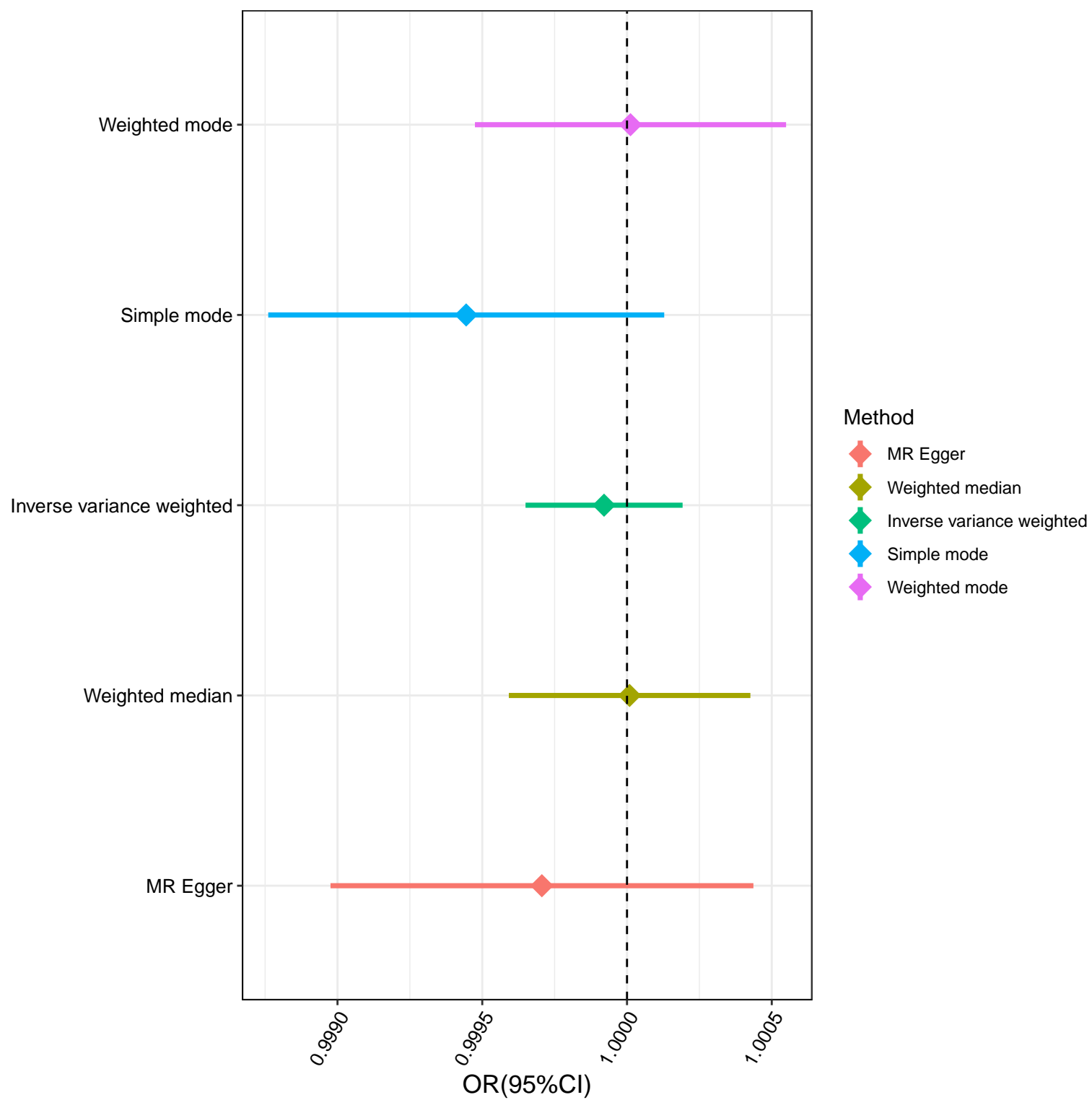

Supplement: Supplementary Figure 2 — Forest plot to visualize causal effects of variation in TSH on HCC. Presented odds ratios (OR) and confidence intervals (CI) correspond to the effects of TSH on HCC. The results of Mendelian Randomization (MR) analyses using various analysis methods (MR-Egger, Weighted median, Inverse variance weighted, Simple mode, and Weighted mode) are presented for comparison. [file DataSheet_2.pdf]

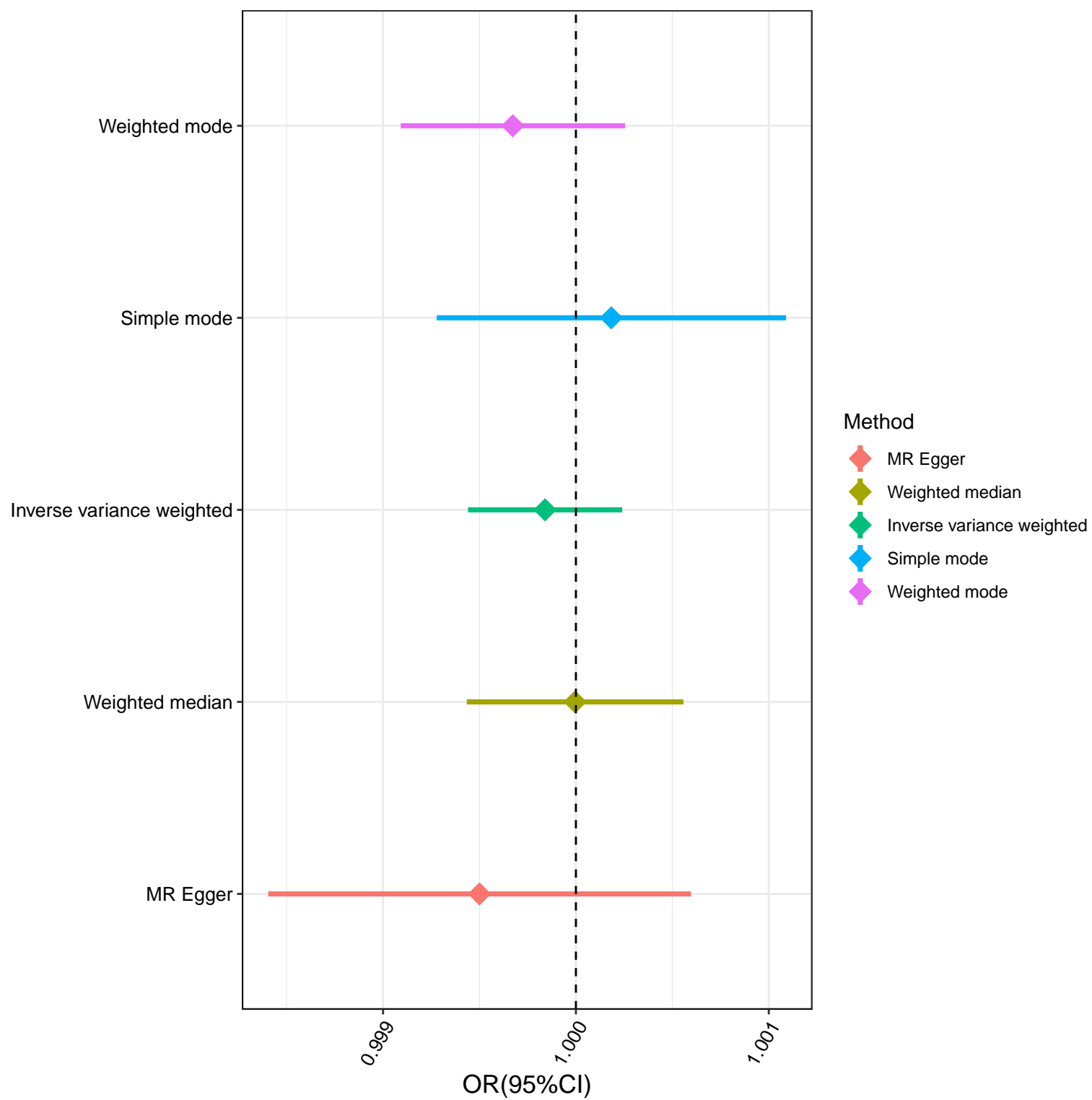

Supplement: Supplementary Figure 3 — Forest plot to visualize causal effects of variation in FT4 on HCC. Presented odds ratios (OR) and confidence intervals (CI) correspond to the effects of FT4 on HCC. The results of Mendelian Randomization (MR) analyses using various analysis methods (MR-Egger, Weighted median, Inverse variance weighted, Simple mode, and Weighted mode) are presented for comparison. [file DataSheet_3.pdf]
