# Supplementary material for: Hypothyroidism has a protective causal association with hepatocellular carcinoma: A two-sample Mendelian randomization study
Source: Front Endocrinol (Lausanne). 2022 Sep 30;13:987401. doi: 10.3389/fendo.2022.987401 (PMC9562779; doi:10.3389/fendo.2022.987401)
Supplement: Supplementary file 4 [file DataSheet_4.pdf]

Table S1. SNPs associated with hypothyroidism

SNP	chr	effect_allele	other_allele	*P*-value	beta (β)	se	EAF	R^2	F.statistic
rs10036386	5	T	C	1.75E-09	0.0032141	0.00053396	0.381915	0.010366001	43.58938376
rs1032129	8	C	A	2.67E-08	-0.00302426	0.000543772	0.35438	-0.009577811	39.47940305
rs10424978	19	A	C	7.21E-15	-0.0041633	0.00053505	0.59984	-0.01339949	55.02393889
rs1045853	14	C	A	1.1E-09	-0.00337461	0.000553686	0.656897	-0.010495908	43.22445985
rs1088898	17	T	G	4.22E-08	0.00338586	0.00061771	0.768046	0.009439499	39.65628429
rs10956412	8	C	A	5.84E-13	-0.00508727	0.000706129	0.163144	-0.012406556	50.99650059
rs111618453	13	A	G	4.33E-23	0.00577379	0.000583397	0.272172	0.017041885	72.14840199
rs11258303	10	A	C	5.59E-10	0.00371265	0.000598646	0.746995	0.010680045	44.92420452
rs11571297	2	C	T	5.34E-61	-0.00859242	0.000521363	0.4907	-0.028371633	114.8096385
rs11675342	2	T	C	1.67E-18	0.00461109	0.000525295	0.422209	0.015115921	63.86951288
rs11706511	3	G	A	2.47E-09	0.00465932	0.000781286	0.126089	0.010270072	43.18181572
rs11783023	8	T	C	1.06E-08	-0.00329795	0.000576457	0.720606	-0.009852353	40.6000155
rs12582330	12	T	G	1.1E-12	-0.00416475	0.00058512	0.728871	-0.012257313	50.39047527
rs12980063	19	G	A	1.74E-10	-0.00339664	0.000532123	0.391004	-0.010992467	45.24716149
rs13090803	3	T	G	2.09E-15	0.0050706	0.00063893	0.212032	0.013666252	57.65933508
rs13145888	4	C	T	2.45E-21	-0.00601133	0.000633817	0.212492	-0.016331748	66.87153284
rs1534430	2	T	C	3.99E-14	-0.00402779	0.000532667	0.389593	-0.013021413	53.49135393
rs1549142	19	T	C	3.9E-11	0.0040914	0.000619147	0.228817	0.011379782	47.90143993
rs16903097	8	G	T	3.97E-09	-0.00452308	0.00076851	0.130854	-0.010135527	41.75522254
rs17020110	1	C	T	2.07E-12	0.00413049	0.00058753	0.267645	0.012106639	50.99852815
rs1790604	18	G	A	5.49E-10	-0.00322907	0.000520432	0.536379	-0.010684952	43.9947532
rs2111485	2	G	A	7.84E-13	0.00380166	0.000530651	0.609397	0.012337158	51.98170601
rs2123340	9	A	G	7.76E-12	-0.00373671	0.000546057	0.651292	-0.011784337	48.46869387
rs221786	7	C	T	1.53E-09	0.00494967	0.000819327	0.886238	0.010403498	43.74871826
rs229540	22	G	T	8.83E-22	0.00503721	0.000525234	0.423587	0.016514366	69.87760093
rs2412974	22	T	C	8.01E-09	-0.00311955	0.000540792	0.358692	-0.009934	40.93316063
rs244672	5	T	C	4.84E-09	-0.00462791	0.000790748	0.877485	-0.010078796	41.52383909
rs2745803	20	G	A	9.31E-09	-0.00368215	0.000641164	0.207698	-0.009889971	40.75351359
rs28157	5	T	G	4.16E-10	-0.00349175	0.000558853	0.316822	-0.010759803	44.29966708
rs2823272	21	A	T	2.03E-10	-0.00355403	0.000558859	0.31568	-0.010951578	45.08067921
rs28418426	6	C	T	3.06E-58	0.00917276	0.000570025	0.5296	0.027702778	118.5682421
rs3184504	12	C	T	1.1E-81	-0.0099309	0.000518629	0.517816	-0.032959431	132.7824069
rs3775291	4	T	C	1.05E-12	-0.00403311	0.000566167	0.297729	-0.012267237	50.43077767
rs3850765	10	C	T	2.76E-09	0.00313025	0.000526496	0.585106	0.010238706	43.04856985
rs4263621	6	A	G	1.48E-08	0.00295553	0.000521765	0.537567	0.009754919	40.99445163
rs4276275	4	T	C	5.84E-11	0.00340427	0.000519895	0.472519	0.011276249	47.46066159
rs4409785	11	C	T	4.31E-21	0.00647971	0.00068747	0.171755	0.016230374	68.65611266
rs4444866	4	T	C	2.85E-08	-0.00322868	0.000581693	0.278846	-0.009558621	39.40104985
rs60600003	7	G	T	7.19E-09	0.00500327	0.000864647	0.100946	0.009965003	41.88620376
rs61759532	17	T	C	1.26E-11	0.00419164	0.000618829	0.246518	0.011664542	49.11423738
rs62076510	17	G	T	3.46E-13	0.00520033	0.000714773	0.163945	0.01252889	52.79980126
rs6426808	1	A	G	3.13E-10	0.00327227	0.000520025	0.509924	0.010836357	45.58891678
rs654537	6	A	G	2.13E-29	0.00599281	0.000532298	0.611191	0.019385543	82.26664271
rs6584277	10	G	A	8.79E-10	-0.0031819	0.000519066	0.525655	-0.01055659	43.47174922
rs66749983	13	T	A	1.39E-10	0.00360186	0.000561314	0.310435	0.011050409	46.49950092
rs6679677	1	A	C	1.07E-122	0.0201022	0.000852993	0.102573	0.040553153	175.8926969
rs6833591	4	G	A	6.1E-09	-0.00317388	0.000545888	0.347415	-0.010012651	41.25403012
rs683763	10	T	G	2.57E-08	0.00309064	0.000555044	0.321202	0.009589257	40.29152529
rs6992869	8	C	T	2.56E-08	0.00299144	0.000537158	0.372952	0.009590521	40.29688695
rs705702	12	G	A	1.96E-11	0.00367966	0.00054845	0.33759	0.011553819	48.64258483
rs7090530	10	A	C	2.94E-15	0.00418704	0.000530431	0.603596	0.013593209	57.34691268
rs71508903	10	T	C	3.21E-21	0.00625717	0.000661691	0.193712	0.016283548	68.88477083
rs736374	11	A	G	2.48E-14	0.00412144	0.000540637	0.363007	0.013127732	55.35703964
rs7441808	4	G	A	3.53E-10	0.00354074	0.000564386	0.302384	0.010803796	45.45043323
rs7582694	2	G	C	2.61E-29	-0.00697507	0.000620534	0.773961	-0.01935466	79.01405081
rs761357	6	T	A	6.81E-10	0.00331651	0.000537459	0.376767	0.010626625	44.69708909
rs7649344	3	C	T	1.23E-08	-0.00296836	0.000521217	0.458522	-0.009807561	40.41722601
rs7768019	6	G	C	1.31E-41	-0.00812346	0.000601065	0.246831	-0.023269439	94.63243256
rs7905731	10	C	T	2.66E-09	-0.00314417	0.000528315	0.594309	-0.010248827	42.2172484
rs8008961	14	T	C	5.54E-09	-0.00336955	0.000577942	0.27975	-0.010040369	41.36709618
rs8043085	15	T	G	4.47E-12	0.00425081	0.000614119	0.233733	0.011919889	50.20236275
rs8054578	16	G	A	8.95E-09	-0.00358256	0.000623096	0.776427	-0.009901503	40.80056821
rs853303	8	G	A	2.3E-12	-0.00374837	0.000534335	0.621756	-0.012080393	49.67182751
rs911760	9	A	C	5.1E-10	0.00418051	0.000672507	0.207503	0.010705119	45.03081629
rs925489	9	T	C	1.52E-71	0.00985707	0.000550975	0.668458	0.030795965	132.2277799
rs926103	1	C	T	4.92E-08	-0.00298868	0.000547963	0.652112	-0.009392754	38.72370048
rs9272426	6	G	A	7.96E-121	0.0123738	0.000529183	0.460274	0.04023734	174.4654816
rs9277569	6	T	C	1.36E-23	0.00840785	0.000839758	0.106947	0.017240512	73.00406064
rs933243	6	A	C	3.9E-24	-0.00557022	0.000549604	0.33412	-0.0174518	71.37900408
rs9497965	6	T	C	5.19E-12	0.00364705	0.000528511	0.408708	0.011883406	50.04686039
rs9815073	3	A	C	1.94E-35	-0.00705726	0.000567968	0.347569	-0.021394234	87.16606584
