# Supplementary material for: Hypothyroidism has a protective causal association with hepatocellular carcinoma: A two-sample Mendelian randomization study
Source: Front Endocrinol (Lausanne). 2022 Sep 30;13:987401. doi: 10.3389/fendo.2022.987401 (PMC9562779; doi:10.3389/fendo.2022.987401)
Supplement: Supplementary file 5 [file DataSheet_5.pdf]

**Table S2.** SNPs associated with TSH									
SNP	chr	effect_allele	other_allele	*P*-value	beta (β)	se	EAF	R2	F.statistic
rs10116772	9	A	C	1.655E-11	0.000121407	0.0061	0.4588	0.029046447	46.37055193
rs10125444	9	A	G	1.599E-09	2.50E-05	6.30E-03	0.5169	-0.025811309	39.00230272
rs10401485	19	T	C	1.158E-15	-1.89E-06	6.30E-03	0.4089	-0.034941932	50.07032894
rs1045475	16	A	G	2.684E-09	8.36E-05	8.20E-03	0.364	0.025586382	40.70176521
rs10799824	1	A	G	4.947E-39	-3.12E-05	8.50E-03	0.1726	-0.057875461	79.69090284
rs10948107	6	A	G	1.047E-24	-1.22E-05	7.10E-03	0.1886	-0.044085741	65.45000539
rs117300236	17	A	G	1.222E-09	-3.56E-05	1.12E-02	0.1474	0.026857622	40.47806798
rs11857151	15	C	G	9.825E-21	-4.31E-05	6.50E-03	0.7166	0.040245799	64.99923425
rs1203933	20	T	C	6.486E-12	-5.93E-05	7.20E-03	0.2775	-0.029494416	44.40823492
rs12284404	11	A	G	2.477E-22	1.56E-05	6.90E-03	0.1722	-0.041453324	61.69745619
rs1265091	6	T	C	3.195E-11	8.12E-05	8.60E-03	0.1571	0.030402546	42.65738557
rs1479560	5	T	G	2.039E-16	-3.86E-05	8.80E-03	0.3516	-0.035542288	52.0111715
rs1479567	5	A	G	2.99E-117	4.59E-06	6.20E-03	0.6332	0.100033657	168.4375712
rs214334	1	A	G	4.246E-16	3.69E-05	6.90E-03	0.7317	-0.036229384	50.92767718
rs2396083	6	C	G	1.187E-40	-2.53E-05	6.90E-03	0.6369	0.057132714	93.92517151
rs28559861	14	T	G	8.677E-14	-4.87E-05	7.10E-03	0.6346	0.032203245	51.57784312
rs2919373	8	T	C	1.655E-14	2.81E-05	7.60E-03	0.2244	-0.032793289	49.21746881
rs3008043	6	A	T	2.903E-48	6.82E-05	6.60E-03	0.2843	-0.063335189	89.15355434
rs30227	16	T	C	7.586E-14	-2.16E-05	6.30E-03	0.6245	-0.031866969	47.87014631
rs3813580	16	C	G	4.641E-42	9.48E-05	6.50E-03	0.2807	-0.058008744	84.98688482
rs3885190	8	T	C	8.618E-09	-1.47E-05	8.00E-03	0.1223	-0.024617681	37.24200058
rs4445669	11	T	C	5.758E-11	2.80E-05	6.10E-03	0.6213	-0.027922056	42.10512022
rs4793440	17	A	G	3.83E-14	-1.95E-05	6.10E-03	0.7147	-0.032348692	48.5711093
rs4933466	10	A	G	5.134E-10	7.97E-05	6.30E-03	0.5513	0.026900195	42.84950007
rs59381142	3	A	G	1.696E-14	4.46E-05	7.60E-03	0.2184	-0.033433194	48.0762923
rs6724363	2	T	G	1.907E-31	1.69E-05	6.90E-03	0.6026	0.051389242	80.50286243
rs7156872	14	C	G	2.78E-18	-4.92E-05	7.60E-03	0.2125	0.037640938	60.62768814
rs73398264	15	T	C	4.429E-33	8.05E-06	6.90E-03	0.8283	0.051187567	83.62417245
rs75125154	1	A	G	5.411E-20	6.00E-05	1.14E-02	0.8576	-0.040550935	56.76574625
rs7695810	4	C	G	2.522E-51	5.33E-05	7.60E-03	0.8104	0.066067653	105.1236964
rs7845302	8	A	C	1.951E-09	2.76E-05	6.60E-03	0.5911	-0.02574325	38.90204356
rs79085669	10	A	G	9.134E-10	3.90E-07	6.60E-03	0.1937	-0.026327923	39.76291004
rs930542	3	A	G	9.259E-11	-3.30E-05	7.00E-03	0.3059	0.027703188	44.1650375
rs9511147	13	C	G	1.813E-11	-2.07E-06	6.50E-03	0.7857	0.029041041	46.36166439
rs9915657	17	T	C	1.906E-18	5.54E-05	6.10E-03	0.4405	-0.037475572	55.99098423
