# Supplementary material for: Hypothyroidism has a protective causal association with hepatocellular carcinoma: A two-sample Mendelian randomization study
Source: Front Endocrinol (Lausanne). 2022 Sep 30;13:987401. doi: 10.3389/fendo.2022.987401 (PMC9562779; doi:10.3389/fendo.2022.987401)
Supplement: Supplementary file 6 [file DataSheet_6.pdf]

**Table S3.** SNPs associated with FT4									
SNP	chr	effect_allele	other_allele	*P*-value	beta (β)	se	EAF	R^2	F.statistic
rs10119187	9	T	C	4.11E-09	-2.05E-05	0.0085	0.8118	0.026333511	111.0136653
rs10759944	9	A	G	1.37E-29	3.27E-05	0.0068	0.2157	-0.050883435	198.7466268
rs10986291	9	A	T	2.06E-11	4.87E-05	0.0069	0.7152	0.030086878	127.3274906
rs11626434	14	C	G	4.08E-17	3.17E-05	0.0069	0.3741	0.038038815	162.310764
rs182416191	4	A	C	4.81E-24	5.05E-05	0.0114	0.03617	0.046649285	192.8774133
rs2235544	1	A	C	4.20E-101	-7.84E-05	0.0065	0.4432	0.095694586	434.3603082
rs4842131	9	T	C	7.68E-44	2.00E-07	0.0075	0.5023	-0.065179404	228.4362396
rs4899764	14	T	G	5.41E-15	0.000120168	0.0068	0.3191	-0.035291459	139.9216372
rs56340915	3	A	G	2.71E-10	5.53E-05	0.01	0.90201	0.028416846	120.0532159
rs6471863	8	A	G	1.09E-10	2.62E-07	0.0095	0.3288	-0.028963918	115.5407153
rs78677597	6	A	C	4.88E-19	-4.22E-05	0.0082	0.8837	-0.040295011	158.9910441
rs9356988	6	A	G	3.56E-12	-5.84E-06	0.0073	0.3901	-0.031459675	125.1929515
